# In silico characterization of the motor subunit of the e.coli. restriction-modification system EcoR1241

**DOI:** 10.1186/1758-2946-5-S1-P2

**Published:** 2013-03-22

**Authors:** Dhiraj Sinha, Morteza Khabiri, David Reha, Rudiger Ettrich

**Affiliations:** 1 Institute of Nanobiology and Structural Biology of GCRC, Academy of Sciences of the Czech Republic; 2 Faculty of Sciences, University of South Bohemia, Nove Hrady, Cz-37333, Czech Republic

## 

Type1 restriction modification system are intriguing multifunctional multisubunit molecular motors that can catalyze both restriction and modification activity. The type 1 RM enzymes binds to its target sequence and its activity as an endonuclease or methyltransferase is determined by the methylation state of the target sequence. If the target sequence is unmodified, the enzyme while bound to its target site is believed to translocate or pull the DNA towards itself simultaneously in both directions in an ATP dependent manner.

The crystal structure of the motor subunit R has been determined by our group but the molecular mechanism by which these enzymes translocate and cleave the DNA is not fully understood.

Our current research effort focuses on full-length three-dimensional structures of the R-subunit, utilizing computational and bioinformatics methods. Modelling of missing loops of residues of the crystal structure is done by Modeller9v2. Optimization of intersubunit contacts is performed by energy minimization followed by molecular dynamics simulations in solution at 300K. The dynamic behavior of WT and mutant holo and apo systems is explored by molecular dynamics simulation in GROMACS using the AMBER99SB force field. Conformational changes connected to coupling of translocation and endonuclease activity are observed and QM/MM methods are applied to calculate the contribution of the residue to the overall binding energy of ATP in the binding pocket.
